# Weekend effect on the incidence and outcomes of cardiac surgery associated - acute kidney injury

**DOI:** 10.1186/s12872-023-03431-4

**Published:** 2023-10-27

**Authors:** Qiwen Xie, Ziyan Shen, Mingzhen Pan, Yang Li, Zhe Luo, Chunsheng Wang, Linxi Zhao, Yi Fang, Xiaoqiang Ding, Jie Teng, Jiarui Xu

**Affiliations:** 1 Department of Nephrology, Zhongshan Hospital (Xiamen), Fudan University, Nephrology Clinical Quality Control Center of Xiamen, No.668 Jinhu Road, Xiamen, 361006 Fujian China; 2 Department of Nephrology, Zhongshan Hospital, Fudan University; Kidney and Dialysis Institute of Shanghai; Kidney and Blood Purification Laboratory of Shanghai, No. 180 Fenglin Road, Shanghai, 200032 China; 3 Department of Nephrology, The Second People’s Hospital of Kashgar, Xinjiang, 844000 China; 4 Department of Critical Care Medicine, Zhongshan Hospital, Fudan University, No. 180 Fenglin Road, Shanghai, 200032 China; 5 Department of Cardiac Surgery, Zhongshan Hospital, Fudan University, No. 180 Fenglin Road, Shanghai, 200032 China; 6 Hopewell Eye Associates, 84 East Broad St, Hopewell, NJ 08525 USA

**Keywords:** Cardiac surgery, Acute kidney injury, Weekend, Short-term outcome, Emergency surgery

## Abstract

**Background:**

The effects of surgical day (workdays or weekends) on occurrence and outcome of cardiac surgery associated -acute kidney injury (CSA-AKI) remains unclear. This study aimed to compare the incidence and short-term outcomes of CSA-AKI in patients undergoing surgery on workdays and weekends.

**Materials and methods:**

Patients who underwent cardiac surgery from July 2020 to December 2020 were retrospectively enrolled in this study. These patients were divided into a weekend group and workday group. The primary endpoint was the incidence of CSA-AKI. The secondary endpoints included renal function recovery and in-hospital mortality. The logistic regression model was used to explore the risk factors for CSA-AKI. Stratification analysis was performed to estimate the association between CSA-AKI and weekend surgery stratified by emergency surgery.

**Results:**

A total of 1974 patients undergoing cardiac surgery were enrolled. The incidence of CSA-AKI in the weekend group was significantly higher than that in the workday group (42.8% vs. 34.7%, *P* = 0.038). Further analysis of patients with CSA-AKI showed that there was no difference in renal function recovery between the workday AKI group and weekend AKI group. There was no difference in in-hospital mortality between the weekend group and workday group (3.6% vs. 2.4%, *P* = 0.327); however, the in-hospital mortality of the weekend AKI group was significantly higher than that of the workday AKI group (8.5% vs. 2.9%, *P* = 0.014). Weekend surgery and emergency surgery were independent risk factors for CSA-AKI. The multiplicative model showed an interaction between weekend surgery and emergency surgery; weekend surgery was related to an increased risk of AKI among patients undergoing emergency surgery [adjusted OR (95% CI): 1.96 (1.012-8.128)].

**Conclusions:**

The incidence of CSA-AKI in patients undergoing cardiac surgery on weekends was significantly higher compared to that in patients undergoing cardiac surgery on workdays. Weekend surgery did not affect the in-hospital mortality of all patients but significantly increased the mortality of AKI patients. Weekend surgery and emergency surgery were independent risk factors for CSA-AKI. Weekend emergency surgery significantly increased the risk of CSA-AKI.

**Supplementary Information:**

The online version contains supplementary material available at 10.1186/s12872-023-03431-4.

## Introduction

Cardiac surgery-associated acute kidney injury (CSA-AKI) is a common clinical complication that is associated with increased mortality and morbidity [1]. In the world, more than 2 million cardiac surgeries are performed every year. The incidence of AKI after cardiac surgery varies from 5 to 42% [2], and CSA-AKI ranks as the second most common cause of AKI in intensive care units (ICUs) next to sepsis [3]. The development of CSA-AKI not only increases the cost, time and care in ICUs and hospitals, but further imposes a huge burden of potential chronic renal impairment in patients in both developed and developing countries [1].

The number of patients undergoing cardiac surgery on weekends is increasing because of a growing patient population with rising cardiovascular risks and compounded emergencies. However, in practice, due to limited medical staff and resources, there is a relative inequity in staff allocation and technical levels for post-operative monitoring throughout surgery during workdays vs. weekends. Although several studies reported the outcomes of weekend and workday admissions and surgeries, the effects of surgical day (workday or weekend) on incidence and outcome of CSA-AKI and interrelated adverse events remain unclear [4–7]. Therefore, it is imperative to analyze the CSA-AKI patterns on workdays and weekends to facilitate the optimization of clinical treatment flow and reasonable staffing.

We hypothesized that patients who underwent cardiac surgery on weekends would have a higher incidence of CSA-AKI and worse short-term outcomes compared with those of patients who underwent cardiac surgery on workdays, and the day of cardiac surgery (workday or weekend) is a key factor in CSA-AKI.

## Materials and methods

### Patient selection

**Fig. 1:**
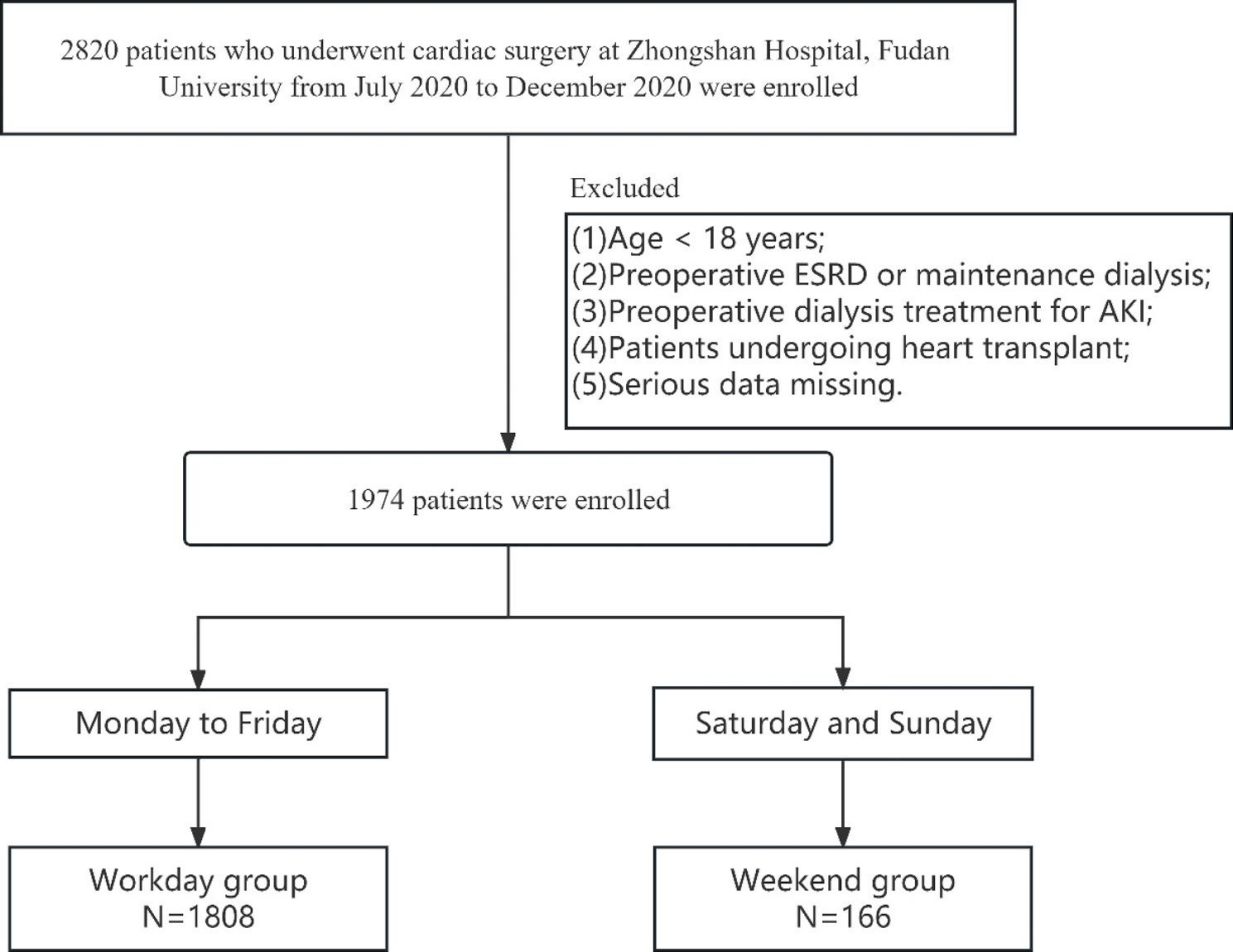
Flow chart of the study protocol

A total of 2820 patients who underwent cardiac surgery from July 2020 to December 2020 in our hospital were all retrospectively enrolled in this study, observational period defined as the duration from the date of admission to the date of discharge for each patient included in the study. Exclusion criteria included: (1) Age < 18years; (2) Preoperative end-stage renal disease (ESRD) or maintenance dialysis; (3) Preoperative dialysis treatment for AKI; (4) Patients undergoing heart transplantation; and (5) Serious data missing. After excluding 846 cases, a total of 1974 patients were included in the study (Fig. 1).

### Groups and endpoints

According to the day of surgery, patients were divided into a workday group(Monday to Friday)or weekend group (Saturday and Sunday). The primary endpoint events were the incidence of AKI, severe AKI and AKI requiring renal replacement therapy (AKI-RRT) after cardiac surgery. The secondary endpoints were renal function recovery, length of hospitalization, length of ICU stay, and in-hospital mortality.

### Data collection and evaluation

The following perioperative data were collected: age, gender, body mass index (BMI), preoperative comorbidity including hypertension and diabetes, heart functional grading according to New York Heart Association (NYHA), coronary angiography, preoperative laboratory indicators including serum creatinine (SCr), serum uric acid, urinary protein, estimated glomerular filtration rate (eGFR) using equations developed by the Chronic Kidney Disease Epidemiology Collaboration (CKD-EPI) for adults ≥ 18 years [8], duration between RRT and surgery, length of hospitalization, length of ICU stay, hospitalization expenses, operation type, cardiopulmonary bypass (CPB) time and aortic occlusion time, and risk factor score for cardiovascular surgery in Europe (EURO Score) [9].

### Definitions

AKI was diagnosed and graded according to the Kidney Disease: Improving Global Outcomes (KDIGO) guideline [10]. Severe AKI was defined as AKI stage 2 and 3. Renal function recovery was defined according to the following: (1) complete renal recovery was defined as dialysis independent and a return of SCr at discharge to within 1.5-fold the baseline; (2) partial recovery was defined as dialysis independent but failure of the SCr to meet the criteria for complete renal recovery; (3) no recovery was defined as dialysis dependent or failure of the SCr to decline compared with SCrmax [11]. Systemic inflammatory response syndrome (SIRS) was identified according to the 2001 SCCM/ESICM/ACCP/ATS/SIS International Sepsis Definitions Conference and International Guidelines for Management of Sepsis and Septic Shock [12]. Low cardiac output syndrome (LCOS) was defined as a decrease in cardiac index (CI) to < 2.0 L/min/m^2^ and systolic blood pressure < 90mmhg, accompanied by signs of tissue hypoperfusion [13].

### Statistical analysis

Statistical analysis was performed using SPSS software (version 20.0, IBM, Armonk, NY, USA). Normality test data are represented by histogram. Normally distributed values are presented as the mean ± standard deviation, and the independent *t* test was used to compare differences between groups. Non-normal distributed values are presented as the median and interquartile (25-75%), and the Wilcoxon rank-sum test was used for comparison between groups. The logistic regression model was used to explore the risk factors for CSA-AKI. Candidate variables with a *P* value < 0.05 on univariable analysis were included in the multivariable logistic regression model. The odds ratio (OR) of AKI predictors was calculated using a 95% confidence interval (CI). Stratification analysis was performed to estimate the association between CSA-AKI and weekend surgery stratified by emergency surgery. Interactions between weekend and emergency surgery were estimated by the product terms in the logistic regression model. A *P* value < 0.05 was considered statistically significant.

## Results

### Clinical characteristics and AKI incidence

A total of 1974 patients underwent cardiac surgery and were included in this study; 1118 (56.6%) were male, and the average age was 56.1 ± 12.9 years. A total of 166 (8.4%) procedures were performed during the weekends, and 1808 (91.6%) procedures were performed during workdays. The two groups were similar regarding perioperative clinical characteristics including gender, age, preoperative comorbidities, preoperative laboratory indicators and the type of surgical procedure. The proportion of emergency surgery on the weekends was higher than that on workdays (8.4% vs. 4.9%, *P* = 0.047) (Table 1).

**Table 1:** Clinical characteristics and AKI incidence of workday and weekend groups

	All	Workday group	Weekend group	*P* value
	N = 1974	N = 1808	N = 166	
Clinical characteristics				
Male [n (%)]	1118(56.6%)	1027(56.8%)	91(54.8%)	0.622
Age (y)	56.1 ± 12.9	56.2 ± 12.9	55.4 ± 12.4	0.453
Hypertension [n (%)]	719(36.4%)	658(36.4%)	61(36.7%)	0.928
Diabetes [n (%)]	244(12.4%)	226(12.5%)	18(10.8%)	0.535
NYHA III-IV [n (%)]	1494(75.7%)	1369(75.7%)	125(75.3%)	0.904
Preoperative SCr (µmol/L)	82.4 ± 32.4	82.2 ± 32.1	84.5 ± 35.6	0.38
Preoperative eGFR < 60 (1.73 ml/min/m^2^) [n (%)]	206(10.4%)	183(10.1%)	23(13.9%)	0.132
Preoperative uric acid(µmol/L)	355.5 ± 110.6	355.3 ± 110.7	357.6 ± 109.7	0.806
Proteinuria [n (%)]	103(5.2%)	93(5.1%)	10(6%)	0.625
Emergency surgery [n (%)]	102(5.2%)	88(4.9%)	14(8.4%)	**0.047**
Preoperative EURO Score	3.5 ± 3.1	3.5 ± 3.1	3.7 ± 3.2	0.403
Surgery category:				0.095
Valves	1040(52.7%)	966(53.4%)	74(44.6%)	
CABG	319(16.2%)	282(15.6%)	37(22.3%)	
Aortic	149(7.5%)	132(7.3%)	17(10.2%)	
Combined surgery	246(12.5)	231(12.8)	15(9.0)	
Others	220(11.1%)	197(10.9%)	23(13.9%)	
CPB time(min)	102.6 ± 43.9	102.7 ± 43.8	102.4 ± 44.5	0.943
Aortic cross-clamp time(min)	60.87 ± 31.8	60.9 ± 31.9	61 ± 30.2	0.96
AKI incidence				
AKI incidence [n (%)]	699(35.4%)	628(34.7%)	71(42.8%)	**0.038**
Severe AKI [n (%)]	159(8.1%)	141(7.8%)	18 (10.8%)	0.167
AKI-RRT [n (%)]	49(2.5%)	44(2.4%)	5(3.0%)	0.347
Duration between RRT and surgery (d)	2[1, 4]	1[1, 4]	2[1,2.5]	0.972
Crude in-hospital mortality [n (%)]	49(2.5%)	43(2.4%)	6(3.6%)	0.327

A total of 699 (35.4%) patients developed AKI. The incidence of CSA-AKI in the weekend group was significantly higher than that in the workday group (42.8% vs. 34.7%, *P* = 0.038). We further analyzed patients in the weekend group and found that the CSA-AKI incidence of those who underwent emergency surgery was significantly higher than that in those who underwent non-emergency surgery (69.2% vs. 40.5%, *P* = 0.045). There was no difference in incidence of severe AKI (10.8% vs. 7.8%, *P* = 0.167) or AKI-RRT (3.0% vs. 2.4%, *P* = 0.347) between the weekend group and workday group. In addition, there was no significant difference in duration of surgery and RRT initiation (2[1,2.5] vs. 1[1, 4] d, *P* = 0.972) (Table 1).

### Short-term outcomes

There was no significant difference in in-hospital mortality between the weekend group and workday group (3.6% vs. 2.4%, *P* = 0.327) (Table 1). Further analysis of AKI patients showed that the in-hospital mortality of the weekend AKI group was significantly higher than that in the workday AKI group (8.5% vs. 2.9%, *P* = 0.014). The proportion of post-operative SIRS was higher in the weekend AKI group than in the workday AKI group (39.4% vs. 28.2%, *P* = 0.048). In terms of the proportion of post-operative LCOS, there was no difference between the two groups. There was no difference regarding the rate of renal function recovery, SCr level at discharge, length of ICU stay and hospitalization expenses between the weekend AKI group and workday AKI group **(**Table 2**)**.

**Table 2:** Short-term outcomes of workday AKI and weekend AKI groups

	Workday AKI group	Weekend AKI group	*P* value
	N = 628	N = 71	
Renal function recovery[n(%)]			0.727
Complete	474(75.5%)	51(71.8%)	
Partial	62(9.9%)	9(12.7%)	
No	92(14.6%)	11(15.5%)	
SCr at discharge (µmol/L)	99.8 ± 73.1	101.5 ± 77.2	0.856
Post-operative LCOS[n(%)]	50(8.0%)	8(11.3%)	0.338
Post-operative SIRS [n(%)]	177(28.2%)	28(39.4%)	**0.048**
Length of hospitalization (d)	14[11,19]	13[10, 17]	**0.011**
Length of ICU stay(d)	2[1, 5]	3[2, 4]	0.091
Hospitalization expenses (10,000 RMB)	16.3 ± 7.5	16.7 ± 11.3	0.698
Crude in-hospital mortality [n(%)]	18(2.9%)	6(8.5%)	**0.014**

### Associations between CSA-AKI and weekend surgery stratified by emergency surgery

The proportion of weekend surgery patients in the AKI group was significantly higher than that in the non-AKI group (10.2% vs. 7.5%, *P* = 0.038, Supplement Table 1). Patients with AKI had a higher proportion of emergency surgery than patients in the non-AKI group, and the difference was statistically significant (*P* = 0.036, Supplement Table 1). In the multivariable regression model (Supplement Table 2), weekend surgery and emergency surgery were independent risk factors for AKI (adjusted ORs and [95% CI]: 1.342 [1.012–1.881] and 1.393 [1.212–2.156], respectively). There was interaction of multiplicative model between weekend surgery and emergency surgery by interaction analysis, and the interactions were significant (*P*_interaction_ = 0.045). Stratification analysis showed that weekend surgery was related to an increased risk of AKI among patients who underwent emergency surgery [adjusted OR (95% CI): 1.96 (1.012-8.128)], whereas the association was not significant among patients with non-emergency [adjusted OR (95% CI): 1.293 (0.909‐1.838), Table 3].

**Table 3:** Associations between CSA-AKI and weekend surgery stratified by emergency surgery

Variables	OR (95%CI)^a^	OR (95%CI)^b^
**Non-emergency surgery**		
Workday surgery	Reference (1.00)	Reference (1.00)
Weekend surgery	1.311(0.935–1.840)	1.293(0.909–1.838)
**Emergency surgery**		
Workday surgery	Reference (1.00)	Reference(1.00)
Weekend surgery	**2.65(1.110–8.558)**	**1.960 (1.012–8.128)**

a: Unadjusted;

b: Adjusted by age, diabetes, history of cardiac surgery, preoperative eGFR < 60ml/min/m^2^, preoperative uric acid, and preoperative proteinuria

## Discussion

In our study, we found that the incidence of CSA-AKI was higher in the weekend group than in the workday group, while there was no significant difference in the occurrence of severe AKI and AKI-RRT between the two groups. According to our study, there was no difference in in-hospital mortality between the weekend and workday groups, whereas further analysis of AKI patients revealed that the in-hospital mortality in the weekend AKI group was much higher compared with that in the workday AKI group. Weekend surgery and emergency surgery were independent risk factors for CSA-AKI. There was interaction in the multiplicative model between weekend surgery and emergency surgery; weekend surgery was related to an increased risk of AKI among patients who underwent emergency surgery.

“Weekend effect” was first analyzed according to the mortality rate in neonatal or perinatal patients, which was higher on weekends than on workdays [14, 15]. Consistent with our study of a higher incidence of CSA-AKI in the weekend group, a retrospective study of 305,853 patients undergoing cardiac, gastrointestinal or vascular surgeries showed that patients who underwent surgery over the weekend were more likely to have serious complications (OR 1.6) including death (2-fold for emergency surgery; 3-fold for selective surgery) than those who underwent surgery on workdays [4]. In contrast, a study of 106,473 patients who underwent cardiac surgery in Sweden showed that short-term and long-term prognosis after cardiac surgery were not affected by surgery on workdays or weekends [16].

“Weekend effect” can be explained in that staffing is relatively insufficient on the weekend, and some of them tend to suffer from fatigue [4]. Furthermore, weekend operations tend to address more serious or emergent conditions. Indeed, our study also found that the proportion of emergency surgeries was significantly higher on weekends. However, in our hospital, the nephrology consultation team is comprised of experienced nephrologists working around the clock seven days a week. This may be the reason that we found no difference in the occurrence of severe AKI and no difference in duration between the operation and RRT initiation between the weekend and workday groups.

In our results, emergency surgery on the weekend further increased the incidence of AKI, which was up to 69.2%. Although there was no difference in in-hospital mortality between the weekend and workday groups, the in-hospital mortality in the weekend AKI group was much higher compared with that in the workday AKI group. Whether emergency surgery performed on the weekend had an effect on primary outcomes varied across studies. A study that enrolled 50,844 patients found no difference in short-term mortality following emergency general surgery on the weekend compared with that on a workday [17]. However, some studies showed diametrically opposite results. Coppolino G et al. found that patients undergoing emergency surgery on the weekend were susceptible to CSA-AKI, and AKI markedly increases mortality risk [18]. Some studies suggested that patients requiring emergency surgery on the weekend were frail, which increased the risk of mortality; however, many investigators attributed it to insufficient hospital staffing and resources, especially the lack of experienced medical experts, which may lead to delayed diagnosis and non-adherence to protocols.

Our study showed that emergency surgery and weekend surgery were independent risk factors for CSA-AKI. Weekend emergency surgery significantly increased the incidence of AKI, and the in-hospital mortality of AKI patients was much higher. Elective surgeries can occur on a weekend, which helps to meet the increasing demand for surgery, but also reduces the risks associated with surgery as much as possible. At present, policymakers tend to reverse-infer the factors in a medical institution that contribute to “weekend effect” and prefer to prioritize improving the human factors (i.e., staffing) because “hardware” conditions are relatively difficult to change. Some medical institutions in China are trying to perform weekend operations to alleviate the growing patient load. Therefore, under the existing hardware resources, weekend operations help to better schedule the number of surgical procedures, speed up bed turnover, and make full use of operating room resources. With equal staffing and reasonable crew rotations throughout the week, “weekend effects” are likely to be lessened or eliminated altogether [4].

The primary strength of this study is that the endpoints in our study are relatively comprehensive. Besides the AKI incidence, we investigated the incidence of all-cause in-hospital mortality and other complications such as LCOS and SIRS as well as the hospitalization expenses and renal function recovery. Second, the sample size is adequate. Third, since there are few studies on the “weekend effect” in China, our study may give rise to rational allocation of medical resources and staffing on weekends and workdays for hospital and government policymakers with use of indigenous data. The limitations of the study include the following: First, because our study was retrospective, confounding factors and choice bias still exist. Second, this is an observational study of a single center; therefore, further multicenter and multidimensional studies are needed for more convincing conclusions. Third, other risk factors for AKI such as use of nephrotoxic medications and infective endocarditis were not considered, and this study lacks long-term follow-up data.

## Conclusion

In conclusion, this study found that the incidence of CSA-AKI in the weekend group was significantly higher than that in the workday group. The in-hospital mortality was much higher in the weekend AKI group compared with that in the workday AKI group. In addition, weekend surgery and emergency surgery were independent risk factors for CSA-AKI. Weekend emergency surgery significantly increased the risk of CSA-AKI. As a complex phenomenon, “weekend effect” may be the result of multiple factors stemming from a variety of roles in different medical institutions.

### Electronic supplementary material

Below is the link to the electronic supplementary material.

**Supplementary Table S1** Comparison of AKI and non-AKI patients after cardiac surgery.

**Supplementary Table S2** Logistic regression of risk factors for CSA-AKI

## Data Availability

The datasets used and/or analysed during the current study are available from the corresponding author on reasonable request.

## Abbreviations

CSA-AKI: Cardiac surgery associated-acute kidney injury
ICU: Intensive care unit
AKI: Acute kidney injury
AKI-RRT: AKI requiring renal replacement therapy
ESRD: End-stage renal disease
BMI: Body mass index
NYHA: New York Heart Association
SCr: Serum creatinine
eGFR: Estimated glomerular filtration rate
CKD-EPI: Chronic kidney disease-epidemiology collaboration
CPB: Cardiopulmonary bypass
EURO Score: Risk factor score for cardiovascular surgery in Europe
KDIGO: Kidney Disease:Improving Global Outcomes
SIRS: Systemic inflammatory response syndrome
LCOS: Low cardiac output syndrome
CAG: Coronary angiography
CABG: Coronary artery bypass grafting
