# Supplementary material for: Weekend effect on the incidence and outcomes of cardiac surgery associated - acute kidney injury
Source: BMC Cardiovasc Disord. 2023 Oct 27;23:524. doi: 10.1186/s12872-023-03431-4 (PMC10612359; doi:10.1186/s12872-023-03431-4)
Supplement: Supplementary file 1 — Supplementary Table S1 Comparison of AKI and non-AKI patients after cardiac surgery. [file 12872_2023_3431_MOESM1_ESM.pdf]

Supplement Table

Table S1 Comparison of AKI and non-AKI patients after cardiac surgery

	AKI N=699	Non-AKI N=1275	*P*
Male [n (%)]	404 (57.8%)	714 (56%)	0.441
Age (y)	58±12.2	55.1±13.2	**<0.01**
BMI (kg/m^2)	23.3±3.4	23.3±3.2	0.688
Hypertension [n (%)]	259 (37.1%)	460 (36.1%)	0.667
Diabetes [n (%)]	71 (10.2%)	173 (13.6%)	**0.028**
NYHA III-IV [n (%)]	533 (76.3%)	961 (75.4%)	0.663
History of cardiac surgery [n (%)]	89 (12.7%)	100 (7.8%)	**<0.01**
Preoperative CAG [n (%)]	400 (57.2%)	690 (54.1%)	0.184
Time between CAG and surgery(d)	3[2,6]	3[1,5]	0.434
Preoperative eGFR <60ml/min/m^2[n(%)]	127 (18.2%)	79 (6.2%)	**<0.01**
Preoperative uric acid (μmol/L)	379.9±122.9	342.3±100.8	**<0.01**
Preoperative Hct (%)	39.7±5.8	39.3±6.4	0.172
Preoperative proteinuria [n (%)]	47 (6.7%)	56 (4.4%)	**0.026**
Emergency surgery[n(%)]	46 (6.6%)	56(4.4%)	**0.036**
Weekend surgery [n (%)]	71 (10.2%)	95 (7.5%)	**0.038**
CPB time (min)	99.6±40.9	104.2±45.2	0.06
Aortic cross-clamp time (min)	59.9±29.2	61.3±33.1	0.443
