# Supplementary material for: Weekend effect on the incidence and outcomes of cardiac surgery associated - acute kidney injury
Source: BMC Cardiovasc Disord. 2023 Oct 27;23:524. doi: 10.1186/s12872-023-03431-4 (PMC10612359; doi:10.1186/s12872-023-03431-4)
Supplement: Supplementary file 2 — Supplementary Table S2 Logistic regression of risk factors for CSA-AKI [file 12872_2023_3431_MOESM2_ESM.pdf]

Table S2 Logistic regression of risk factors for CSA-AKI

Variables	Univariable logistic regression		Multivariable logistic regression	
	OR	95%CI	OR	95%CI
Age(y)	1.019	1.011-1.026	**1.016**	**1.008-1.024**
Diabetes (Y/N)	1.389	1.036-1.861	**1.393**	**1.024-1.895**
Preoperative eGFR<60 ml/min/m^2(Y/N)	3.361	2.495-4.528	**2.215**	**1.594-3.077**
Preoperative uric acid (μmol/L)	1.003	1.002-1.004	**1.002**	**1.001-1.003**
Preoperative proteinuria (Y/N)	1.591	1.177-2.150	1.255	0.915-1.718
History of cardiac surgery (Y/N)	1.714	1.268-2.319	**1.709**	**1.247-2.341**
Weekend Surgery (Y/N)	1.644	1.122-2.342	**1.342**	**1.012-1.881**
Emergency Surgery (Y/N)	1.404	1.017-1.938	**1.393**	**1.212-2.156**
